# Draft Genome Sequence of *Lactobacillus sakei* Strain wikim 22, Isolated from Kimchi in Chungcheong Province, South Korea

**DOI:** 10.1128/genomeA.01296-14

**Published:** 2014-12-11

**Authors:** Hyeong In Lim, Jina Lee, Ja Young Jang, Hae Woong Park, Hak-Jong Choi, Tae-Woon Kim, Mi Ran Kang, Jong-Hee Lee

**Affiliations:** World Institute of Kimchi, Gwangju, Republic of Korea

## Abstract

We report the draft genome sequence of *Lactobacillus sakei* strain wikim 22, a *Lactobacillus* species isolated from kimchi in North Chungcheong Province, South Korea, having 155 contigs with 2,447 genes and an average G+C content of 40.61%.

## GENOME ANNOUNCEMENT

*Lactobacillus sakei* is a Gram-positive and facultatively heterofermentative *Lactobacillus* species that belongs to the lactic acid bacteria (LAB) group. *L. sakei* strains are most frequently isolated from traditional dry sausage, meat products, and fish and are used as starter cultures (1–3). Genome analysis of the representative strain *L. sakei* 23K has revealed a combination of adaptation strategies in the species to grow and survive on meat and meat products (4). Compared to *L. sakei*, other LAB such as *Lactobacillus plantarum* and *Lactobacillus curvatus* have also been identified in microbiota from fermented sausages but not in other meats (5, 6). Moreover, *L. sakei* plays an important role in meat fermentation and in the preservation of fresh meat (7). Interestingly, it is also found in the Korean traditional food kimchi, which has mainly vegetable ingredients (8).

*L. sakei* wikim 22 was originally isolated from kimchi, and the primary taxon was identified by 16s rRNA gene sequencing with 99% sequence homology with *L. sakei* 23K (NR075042.1), *L. sakei* DSM 20017 (NR042443.1), and *L. sakei* NBRC 15893 (NR113821.1), respectively.

Genomic DNA was isolated with the QIAamp DNA extraction kit (Qiagen), and quality was confirmed by the Agilent bioanalyzer high sensitivity chip. Genome sequencing was done using Ion Torrent (Life Technologies) and a 318 chip. After trimming the adaptor sequences, 2,088,720 reads were generated with 85.4% trimming efficiency and an average length of 250.1 bp. The sequence was assembled into 155 contigs; the total read length was 512,726,239 bp with a mean contig length of 14,102 bp. The GC content of the sequence was 40.61%, and the reads were mapped to the references using CLC Genomics Workbench version 7.0.4. The total read length was 312,737,826 nt with an average coverage of 164.03×, and the consensus length was 1,756,717 nt. Optimal syntenic layout of unfinished assemblies (OSlay) was used to validate the assembly results with the reference genomes, and the total number of gaps was 55.

The total sequences (CDSs) were predicted by GenemarkS (9), and tRNAs and rRNAs were identified by RNAmmer and tRNAscan, respectively (10, 11). The RAST genome annotation server was adapted to analyze the *L. sakei* genome sequences, which were found to have 2,281 coding sequences (12). The predicted CDSs were computed with the SEED database by BLASTp for functional annotation. The RAST server identified genes present in *L. sakei* 23K but not in the reported strain genome sequences. About 44 genes are missing in the *L. sakei* 23K genome. Importantly, the phosphotransferase system (PTS), which is a component for the uptaking of sugar for utilizing beta-glucoside specific IIA, IIB, IIC, and BglG families, existed in *L. sakei* strain wikim 22.

### Nucleotide sequence accession numbers.

This whole-genome shotgun project has been deposited at DDBJ/EMBL/GenBank under accession number JRFY00000000. The version described in this paper is version JRFY01000000.
